# Malignant transformation of uterine serosal endometriosis to clear cell carcinoma in a postmenopausal patient: a rare case report

**DOI:** 10.3389/fonc.2026.1865870

**Published:** 2026-06-17

**Authors:** Xiaodong Mi, Tong Lin, Xiaogang Zhu, Qian Chen, Xihan Yang, Purun Zou, Dan Shu, Fei Tuo

**Affiliations:** 1 Department of Obstetrics and Gynecology, People’s Hospital of Xiangxi Tujia and Miao Autonomous Prefecture, First Affiliated Hospital of Jishou University, Jishou, Hunan, China; 2 Department of Obstetrics and Gynecology, the Third Xiangya Hospital of Central South University, Changsha, Hunan, China; 3 Department of Pathology, People’s Hospital of Xiangxi Tujia and Miao Autonomous Prefecture, First Affiliated Hospital of Jishou University, Jishou, Hunan, China

**Keywords:** atypical endometriosis, clear cell carcinoma, EAOC, endometriosis, ovarian cancer

## Abstract

**Background:**

Endometriosis is a prevalent benign condition among women of reproductive age. While the overall rate of endometriosis-associated malignancy is low, the risk of malignant transformation increases significantly in postmenopausal patients, with malignancy primarily occurring in ovarian lesions. Reports of malignancy in postmenopausal endometriosis affecting the serosal surface of the uterus are extremely rare. The clinical features are poorly understood, standardised diagnostic or treatment guidelines are lacking, and the mechanisms underlying this malignancy are unknown.

**Case presentation:**

The following case report details a 62-year-old postmenopausal woman who presented with dull lower abdominal pain. A colour Doppler ultrasound revealed a pelvic mass, and a preoperative CT scan suggested a possible tumour of left ovarian origin; tumour marker tests were negative. Laparoscopic exploration confirmed that the lesion originated from the serosal surface of the posterior uterine wall. Furthermore, no abnormalities were noted in the bilateral adnexa. The surgical intervention undertaken was a total hysterectomy, accompanied by bilateral adnexal resection, pelvic lymphadenectomy, pelvic peritoneal biopsy, and omental biopsy. Postoperative pathology confirmed the presence of endometriosis of the uterine serosa, accompanied by focal clear cell transformation. The patient did not receive adjuvant chemotherapy and was placed under close follow-up; a follow-up examination six months later revealed no abnormalities.

**Conclusions:**

The transformation of uterine serosal surface endometriosis into clear cell carcinoma is an exceptionally rare occurrence. This case demonstrates the coexistence of benign endometriosis, atypical endometriosis and clear cell carcinoma components within a single histological section, providing crucial morphological evidence for investigating the pathological progression of malignant transformations in extra-ovarian endometriosis.

## 1 Introduction

Endometriosis is a benign condition defined as the presence of endometrial tissue outside of the uterine endometrium or myometrium. It leads to chronic inflammation and is a prevalent condition among women of reproductive age, with a prevalence of approximately 10% (1). A close association has been observed between the condition and an elevated risk of clear cell carcinoma and endometrioid carcinoma of the ovary, despite the overall rate of malignant transformation remaining low (2). While the prevalence of endometriosis decreases in postmenopausal women, the risk of malignant transformation rises significantly to 1%–2.5% (3). Furthermore, due to the insidious nature of the disease’s symptoms, it is often missed or misdiagnosed, leading to delayed treatment and potentially worse outcomes for patients. The prevailing focus of contemporary research endeavors pertains to ovarian lesions (4), with a paucity of reports concerning malignancy in rare extra-ovarian sites, such as the uterine serosa. The clinical and pathological characteristics of such cases remain unclear, and there is a lack of standardized diagnostic and treatment guidelines. This report details a rare instance of endometriosis on the uterine serosa that progressed to clear cell carcinoma, with the objective of establishing a foundation for the early identification and clinical management of such rare cases.

## 2 Case presentation

A 62-year-old Chinese woman of postmenopausal age presented at a different hospital one week before consulting the First Affiliated Hospital of Jishou University with a history of dull lower abdominal pain. A colour Doppler ultrasound showed a mixed-echo mass in the left adnexal region that was about 55 × 44 × 32 mm in size. The patient has a medical history that includes hypertension and hyperthyroidism, for which she has been receiving long-term oral medication. Her condition is well controlled. This patient’s menstrual history is as follows: first menstruation occurred at the age of 14; natural menopause occurred at the age of 52; prior to this, regular menstrual cycles were observed; and there is no history of dysmenorrhoea. Reproductive history: The patient has had four pregnancies – one vaginal delivery and three induced abortions. Family history: There is no documented history of malignancy among first-degree relatives. A gynaecological examination was performed. A cystic mass, measuring approximately 50 mm in diameter, was palpable in the left posterior quadrant of the uterus. The mass exhibited clear margins, minimal fixation, and mild local tenderness. Following admission, contrast-enhanced CT scans of the chest, entire abdomen and pelvis were performed, revealing a pelvic mass behind the uterus measuring 52 × 49 mm, of undetermined nature, and considered to be a possible left ovarian cyst or malignant ovarian tumour (see **Figure 1**). Laboratory tests demonstrated that complete blood count, liver and kidney function, and thyroid function were all within normal limits; serum tumour markers Carcinoembryonic Antigen (CEA), Alpha-Fetoprotein (AFP), Cancer Antigen 125 (CA125), Cancer Antigen 19-9 (CA199), and Human Epididymis Secretory Protein 4 (HE4) showed no abnormal elevations.

**Figure 1:**
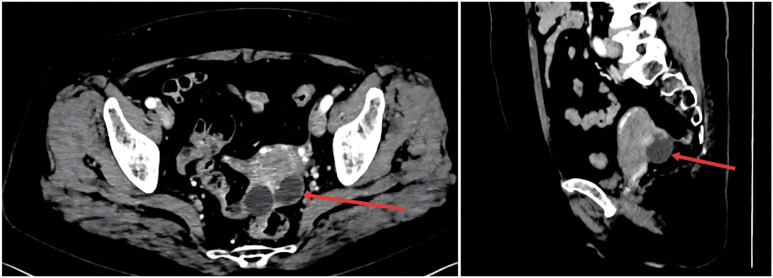
Contrast-enhanced CT of the pelvis (horizontal sections in the intravenous phase and computer-reconstructed sagittal sections) reveals a mass behind the uterus within the pelvis (measuring approximately 52 x 49 mm, indicated by the red arrow), containing fluid.

Preliminary diagnoses of an ovarian tumour or a subserosal uterine fibroid were made on the basis of imaging studies and a physical examination prior to surgery. Surgical treatment was recommended. The patient proceeded to undergo laparoscopic exploration. During the procedure, no obvious abnormalities were observed in the appearance of the ovaries or fallopian tubes on either side. However, a grey-brown cystic-solid mixed mass was identified on the posterior aspect of the uterus, measuring approximately 60 × 60 × 50 mm. The mass was traced to originate from the posterior uterine wall, exhibiting a slightly widened pedicle, as illustrated in **Figure 2**. The mass was completely resected intraoperatively, placed in a specimen bag and removed. Upon incision of the specimen, the cavity was found to be filled with a dark red, chocolate-like viscous fluid. Intraoperative rapid frozen section pathology revealed a diagnosis of focal adenocarcinoma. Following a comprehensive consultation with the patient’s family and the procurement of their consent, a total hysterectomy, a bilateral salpingo-oophorectomy, pelvic lymph node dissection, a pelvic peritoneal biopsy, and an omental biopsy were performed. The lesion was completely resected intraoperatively without rupture. Postoperatively, the patient developed deep vein microthrombosis in the lower limbs; symptoms resolved following anticoagulation and symptomatic treatment, and recovery was satisfactory.

**Figure 2:**
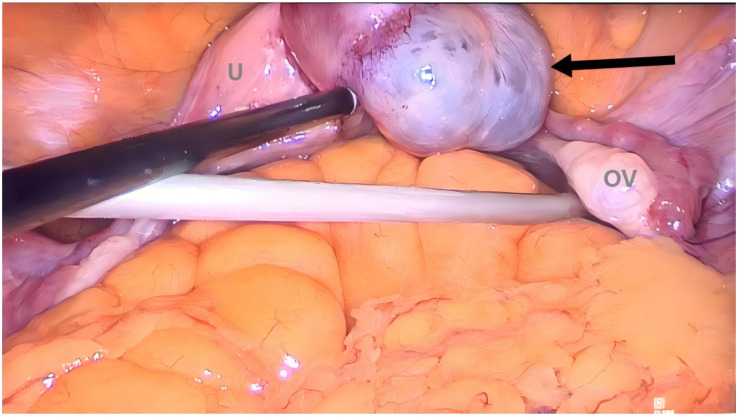
Intraoperative laparoscopic image. The tumour (black arrow) is visualized on the serosal surface of the posterior uterine wall. U, uterus; OV, ovary.

Postoperative pathological findings revealed no evidence of cancerous infiltration in the uterine, bilateral adnexal, pelvic lymph node, pelvic peritoneal, or omental biopsy specimens. Microscopic examination of the mass found on the posterior uterine wall showed multiple endometriotic lesions accompanied by atypical clear cell hyperplasia and focal clear cell carcinoma, measuring approximately 6 × 5 mm. Representative microscopic morphology of the mass are as follows: Under low-power magnification (HE ×2), the black arrow in **Figure 3A** indicates a typical endometriotic lesion, the red arrow indicates atypical clear cells, and the blue arrow indicates extensive clear cell cystic adenomatous changes; in **Figure 3B**, the black arrow indicates an endometriotic lesion, the green arrow indicates clear cell carcinoma tissue, and the yellow arrow indicates uterine smooth muscle tissue. The HE-stained section (**Figure 4A**) shows typical endometriosis morphology; under low magnification, a tubular structure is visible, with the lumen lined by a single layer of columnar epithelium. The cells are arranged in an orderly fashion, with oval nuclei located at the base and pale cytoplasm; HE-stained sections (**Figures 4B, C**) reveal spindle-cell-like morphological changes, accompanied by mild nuclear atypia and simple papillary structures, with no clear stromal invasion. HE-stained sections (**Figures 4D, E**) demonstrate the typical morphology of clear cell carcinoma: tumour cells are polygonal, cuboidal, columnar and flattened, with clear cytoplasm; some cytoplasm is eosinophilic, and characteristic boot-spike-like cells are visible; the histological architecture is predominantly a mixture of tubular-cystic and solid-patchy patterns. Immunohistochemical staining sections (**Figures 4F–J**): Napsin A and PAX-8 were positive, ER and PR were negative, and P53 showed wild-type expression.

**Figure 3:**
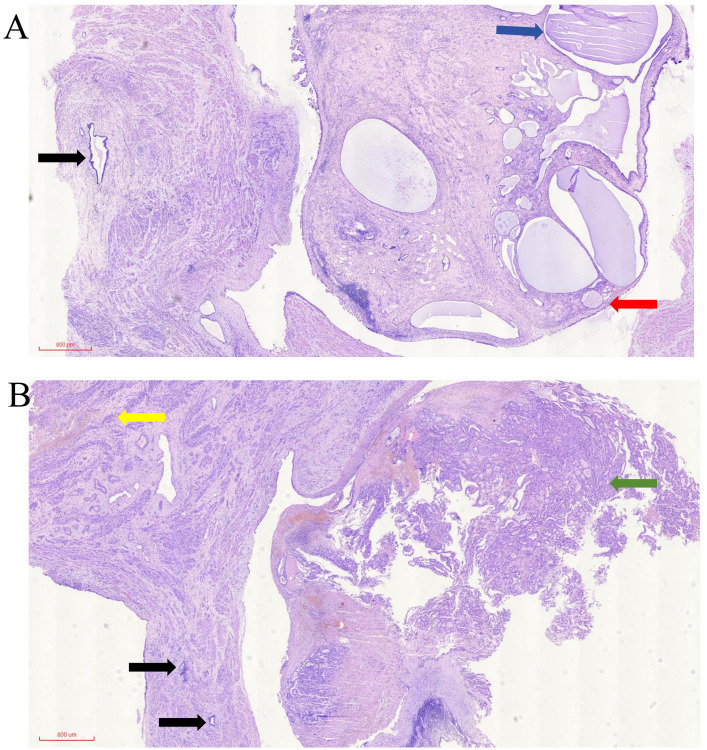
**(A)** shows a section demonstrating both endometriosis and atypical clear cells (HE × 2); **(B)** shows a section demonstrating both endometriosis and clear cell carcinoma (HE × 2). The black arrow indicates a typical endometriotic lesion; the red arrow indicates atypical clear cell cells; the blue arrow indicates a clear cell cystadenoma; the green arrow indicates typical clear cell carcinoma; and the yellow arrow indicates uterine smooth muscle tissue.

**Figure 4:**
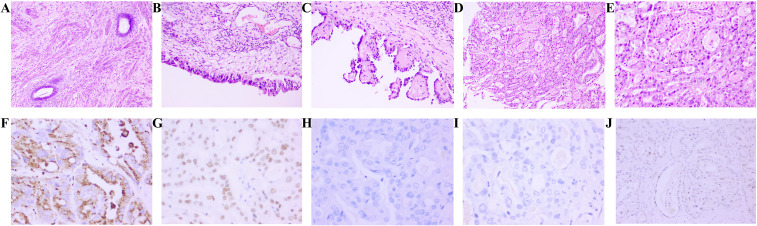
**(A)** Shows an endometriotic lesion (enlarged view for the area indicated by the black arrow in **Figure 3B**) (HE × 200); **(B)** Shows atypical endometrial epithelium (HE × 200); **(C)** Shows atypical clear cell neoplasm with a cystic growth pattern (HE × 400); **(D)** Shows clear cell carcinoma (HE × 200); **(E)** Clear cell carcinoma (HE × 200); **(F)** Napsin A-positive (SP × 400); **(G)** PAX8-positive (SP × 400); **(H)** ER-negative (SP × 400); **(I)** PR-negative (SP × 400); **(J)** Variable nuclear positivity (SP × 400).

The patient was diagnosed with clear cell carcinoma of the uterine serosa. The multidisciplinary team (MDT) proposed an adjuvant treatment plan based on that used for clear cell carcinoma of the ovary. The patient, however, chose not to have adjuvant chemotherapy and was told to go to regular long-term follow-up appointments. Follow-up examinations at three months and six months post-surgery revealed no abnormalities in serum tumour markers (including CEA, AFP, CA125, CA199, and HE4) and abdominal and pelvic ultrasound examinations.

## 3 Discussion

The prevalence of endometriosis among women of childbearing age is approximately 10% (5). The incidence of malignancy in this patient population ranges from 0.5% to 1%, with the majority of cases arising in the ovaries. The most prevalent types of malignancy include endometrioid carcinoma and clear cell carcinoma (2). Following the cessation of menstruation, a decline in oestrogen levels ensues, leading to a decline in the incidence of endometriosis to between 2% and 5% (6, 7). However, the rate of malignancy exhibits a marked increase compared to the premenopausal period, reaching between 1% and 2.5% (3). The sites where malignancy occurs are, in order of frequency, the pelvis, the ovaries and the vaginal stump (8). Research suggests that the cause of postmenopausal endometriosis may be that ectopic lesions can synthesise oestrogen autonomously at the local level (9, 10), thereby maintaining the activity of the lesions in a system-wide environment of low oestrogen levels (11, 12). Concurrently, the use of exogenous oestrogen may further induce or exacerbate the lesions (13–15). The combined effects of persistent local high oestrogen levels, chronic inflammation and genetic abnormalities, coupled with oestrogen-only hormone replacement therapy or oral tamoxifen, further promote the malignant transformation of the lesions (15–17). This case under consideration involves a postmenopausal woman who, five years prior to the onset of symptoms, exhibited no evidence of endometriotic lesions on imaging. However, over a five-year period, an endometriotic lesion originating from the serosal surface of the uterus was discovered, which had developed into clear cell carcinoma. To the best of the knowledge of the present authors, there have been no similar documented cases, neither in the domestic literature nor in the international literature. This report represents the first such documented case worldwide and significantly expands the spectrum of malignant tumours associated with endometriosis. The patient has no history of oestrogen replacement therapy or oral tamoxifen; therefore, it is hypothesised that the primary cause may be the gradual enlargement of small endometriotic lesions on the serosal surface of the uterus that developed prior to menopause, or the gradual enlargement of new endometriotic lesions that arose after menopause, leading to the development of clear cell carcinoma.

This condition is challenging to diagnose definitively prior to surgery, and routine imaging and clinical assessments are easily confused with ovarian tumours. The patient’s preoperative colour Doppler ultrasound and contrast-enhanced CT scans both indicated a cystic-solid mass in the pelvis, suggesting a tumour originating from the left ovary; tumour markers showed no specific elevation, and gynaecological examination also failed to distinguish the origin of the lesion. The ultimate determination of a definitive diagnosis is contingent upon surgical exploration. Intraoperative examination confirmed that the mass originated from the serosal surface of the posterior uterine wall, and the appearance of both ovaries and fallopian tubes was normal. This ruling against primary ovarian tumours based on their anatomical origin provided crucial evidence for further pathological diagnosis.

A substantial corpus of research has now corroborated the hypothesis that women afflicted with endometriosis are predisposed to the development of ovarian cancer (18–20), a condition that is designated as endometriosis-associated ovarian carcinoma (EAOC) (21). The process by which endometriosis becomes malignant remains unclear. It has been hypothesised by some researchers that atypical endometriosis may represent an intermediate stage in the progression from benign to malignant lesions (22–24). This form of atypical endometriosis has also been identified in studies examining clear cell carcinomas associated with endometriosis at caesarean section scar sites (25, 26). This histological section provides a comprehensive and uninterrupted illustration of the progression from benign endometriosis through atypical endometriosis to clear cell carcinoma, thereby offering definitive morphological evidence for the transformation of ectopic endometrium into a malignant tumour. Microscopically, typical endometriotic lesions coexist with cancerous lesions, consistent with Sampson’s criteria for the malignant transformation of endometriosis (27). This provides crucial evidence for elucidating the pathological progression of malignant transformation in extra-ovarian endometriosis and clarifying its pathogenesis.

In this case, the patient underwent laparoscopic exploration for a pelvic mass. Following complete resection of the mass during the procedure, examination revealed that the serous fluid from the lesion had a ‘chocolate-like’ appearance. A rapid intraoperative frozen section examination was performed, suggesting a focal adenocarcinoma associated with endometriosis; consequently, the surgical approach was based on that for early-stage ovarian cancer. At present, there are no standardised clinical guidelines in place regarding the surgical management of malignant transformation of extra-ovarian endometriotic lesions (28). At present, there are no standardised clinical guidelines in place regarding the surgical management of malignant transformation of extra-ovarian endometriotic lesions. In accordance with the findings of contemporary research reports, the preponderance of documented treatment regimens adhere to the management paradigm for endometriosis-related cancers of the ovary, encompassing total hysterectomy with bilateral salpingo-oophorectomy or cytoreductive surgery for ovarian tumours (28–30). For lesions occurring in areas other than the pelvis, the surgical approach is determined by the specific location, such as radical resection of mediastinal tumours (31, 32). There is currently no standardised protocol for adjuvant treatment of malignant extra-ovarian endometriotic lesions. However, based on existing case reports, the treatment regimen most commonly chosen is paclitaxel in combination with carboplatin. For patients with tumours in the gastrointestinal or urinary tracts, the preferred regimens are mFOLFOX-6 (oxaliplatin, leucovorin/calcium folinate, 5-fluorouracil) or MEP (methotrexate, etoposide, cisplatin) (28). In this particular instance, following deliberation by the gynaecological oncology MDT, a treatment plan was formulated in accordance with the established guidelines for clear cell carcinoma of the ovary. However, given the diminutive dimensions of the tumour, the complete surgical excision without rupture or residual disease, and the patient’s refusal of chemotherapy, close follow-up was adopted. A follow-up examination conducted six months after surgery revealed no abnormalities, suggesting that close follow-up may be a viable strategy for patients with very early-stage disease who have no high-risk factors.

This case report has certain limitations regarding clinical management and follow-up: as a single rare case, the sample size is limited, making it difficult to establish generalisable clinical guidelines or determine the optimal management strategy; the current follow-up period of six months is relatively short and insufficient to assess long-term oncological outcomes such as recurrence, disease-free survival and overall survival; furthermore, the safety of close follow-up alone without adjuvant chemotherapy requires longer-term observation and validation; This condition lacks specific preoperative imaging features and diagnostic markers, making it prone to confusion with ovarian-origin tumours and complicating accurate preoperative diagnosis. This study did not perform molecular genetic testing for ARID1A, PIK3CA, PTEN, etc. (33), and is, therefore, unable to further elucidate the mechanisms of malignant transformation in ectopic endometrium at the molecular level; furthermore, the decision not to administer adjuvant chemotherapy was primarily based on patient refusal and clinical judgement regarding the very early stage of the tumour and lacks support from high-level evidence-based medicine; the value of such adjuvant therapy still requires further validation through large-scale studies.

## 4 Conclusion

The transformation of endometriosis on the serosal surface of the uterus in postmenopausal women into clear cell carcinoma is clinically rarely observed and difficult to diagnose preoperatively. Complete surgical resection is considered the optimal treatment modality, with treatment protocols that may be informed by those established for ovarian cancer. Clinicians should exercise caution in cases of malignant transformation of endometriosis in atypical locations, even in the absence of elevated tumour markers. The aetiology of postmenopausal pelvic masses must be meticulously delineated. Postoperative pathology revealed the coexistence of endometriosis, atypical endometriosis and clear cell carcinoma, providing important evidence for studying the pathological progression of endometriosis malignancy outside the ovaries and elucidating its pathogenesis.

## Data Availability

The original contributions presented in the study are included in the article/supplementary material. Further inquiries can be directed to the corresponding author.
